# Anatomical patterns and factors associated with traumatic bowel injury among patients undergoing emergency surgery for abdominal trauma at two teaching hospitals in Uganda

**DOI:** 10.3389/fsurg.2026.1769450

**Published:** 2026-07-22

**Authors:** Tshimanga Tshilumba Esperant, Bienfait Mumbere Vahwere, Virginia Wainaina, Isaac Edyedu, Kiswezi Ahmed

**Affiliations:** 1 Faculty of Clinical Medicine and Dentistry, Department of Surgery, Kampala International University Western Campus, Ishaka-Bushenyi, Uganda; 2 Surgery Department, Hope of Africa University Lubumbashi, Lubumbashi City, Democratic Republic of the Congo

**Keywords:** abdominal trauma, anatomical patterns and Uganda, bowel injury, emergency surgery, factors associated

## Abstract

**Background:**

Bowel injuries represent a growing burden in blunt and penetrating abdominal and it is associated with poor outcome especially in developing countries. However, the anatomical patterns and factors associated are not well understood in the mentioned settings. This study aimed to determine the anatomical patterns and factors associated with traumatic bowel injury among patients undergoing surgery for abdominal trauma at Kampala international University teaching hospital (KIU-TH) and Fort Portal Regional Referral Hospital (FRRH).

**Methods:**

This was a prospective observational study where consecutive sampling was used to select 99 patients of all ages admitted in surgical wards at KIUTH and FRRH with abdominal trauma and undergoing laparotomy during the study period that consented from 30th September to 30th December 2024. During laparotomy, it was confirmed whether there was bowel injury or not and then injury identified was repaired. Analysis was done using STATA version 16. Variables with a *p* value < 0.05 were considered significant.

**Results:**

This study enrolled 99 study participants with abdominal trauma, and 34 of them had bowel injury. The most common part of the bowel injured was the small bowel (76.5%), specifically the jejunum (43.3%). The most frequently performed surgery was primary repair (58.7%). In the multivariate analysis, penetrative trauma (aOR: 3.62, *p* = 0.048) significantly increased the odds of bowel injury. Complications were significantly higher in the bowel injury group (30.6%) compared to those without (12.0%). Two deaths occurred during the study: one patient with bowel injury succumbed to complications of peritonitis and septic shock, while another death occurred in the non-bowel injury group due to liver laceration.

**Conclusion:**

Bowel injury is prevalent in penetrating abdominal trauma and is frequently associated with severe complications such as septic and hypovolemic shock. Strengthening paramedical services and ATLS training is essential to improve hemodynamic stability on arrival and reduce mortality.

## 1 Introduction

Bowel injuries account for 3%–5% of abdominal trauma cases globally, with small bowel injuries being the most frequent (1). Despite extensive global research, limited data exists from Uganda, especially regarding anatomical patterns and associated factors. This study seeks to address that gap by assessing bowel injury patterns among patients undergoing emergency laparotomy following abdominal trauma.

The mortality rate from traumatic bowel injuries ranges widely from 8% to 87%, averaging around 25% (2, 3). Small bowel trauma is most common, with jejunum and ileum injuries being frequent due to anatomical positioning and trauma mechanics (4, 5).

Bowel injury patterns show that small bowel injuries occur in 55%–70% of cases. Within this, the jejunum is injured in 30%–40% and the ileum in 25%–35% of cases. Duodenal injuries are less common, accounting for 3%–10%, especially in the third and fourth segments due to their retroperitoneal location (6). Large bowel injuries occur in 25%–40% of cases, with the transverse colon being the most affected (30%–35%), followed by the right colon (20%–25%) and left colon (10%–15%) (3, 7). Rectal injuries make up 5%–10% of cases and are often due to penetrating trauma (8).

In terms of trauma mechanisms and geographic distribution, blunt trauma predominates in North America and Europe, accounting for approximately 70% of cases, mostly from motor vehicle accidents (6). In Africa and Latin America, penetrating trauma is more common (50%–70%), mainly from gunshots or stabbings (8). Asia presents a mixed pattern, with a high rate of road traffic-related blunt trauma (5).

Demographically, males are predominantly affected, with an average patient age of around 30 years (9). The most common cause of injury is road traffic incidents (60.3%), followed by gunshots and stabbings. Other contributing factors include smoking and alcohol use, both of which weaken the bowel mucosa and increase susceptibility to perforation during trauma (10). Iatrogenic injuries have also risen due to increased use of diagnostic and therapeutic procedures like laparoscopy and endoscopy (11, 12). Additionally, hypotension and hemorrhagic shock can indirectly cause intestinal injury, with elevated levels of intestinal fatty acid-binding protein (I-FABP) correlating well with trauma severity scores (11).

Understanding the anatomical distribution and contributing factors of TBI is essential for effective management. Given the regional variability in injury patterns and trauma mechanisms, local studies are vital for informing treatment protocols and improving outcomes. Further epidemiological research is recommended, especially in low-resource settings like Uganda.

## 2 Materials and methods

### 2.1 Study design

This was a Prospective Observational study carried at two teaching hospitals located in Uganda (Fortportal Regional Referral Hospital (FRRH) and Kampala International University Teaching Hospital (KIU-TH). All the 2 hospitals have surgical departments that consist of general surgeons, senior house officers, medical officers and nurses that manage patients who present with abdominal trauma, bowel injury included. All the 2 hospitals receive patients with abdominal trauma requiring surgery including those with bowel injury.

All patients with abdominal trauma in KIUTH and FRRH with indication for laparatomy.

Patients with abdominal trauma that were found admitted at the two selected hospitals in Western Uganda. Only those patients with abdominal trauma that were found admitted at the two selected hospitals in Western Uganda that satisfied the inclusion criteria as shown below.

### 2.2 Inclusion criteria and exclusion criteria

Patients of all ages admitted in surgical wards at KIUTH and FRRH with abdominal trauma and undergoing laparotomy during the study period that consented. Those that presented after the operation had been done in another hospital were excluded since we were confirming the diagnosis of bowel injury at surgery by direct observation.

### 2.3 Sampling technique

Consecutive sampling was conducted among all patients admitted at the two teaching hospitals' surgical wards with abdominal trauma.

### 2.4 Sample size

The sample size was calculated based on the independent cohort study sample size formula accessed on https://www.openepi.com/SampleSize/SSCohort.htm.

The primary exposure was abdominal trauma. The exposed were traumatic bowel injury, while the unexposed were those without traumatic bowel injury. The odds ratio for traumatic bowel injury was 5.6 (13).

The proportion of those with traumatic bowel injury was set at 40. Using a two-sided significance level (1-alpha) of 0.05, power (1-beta, % chance of detecting) of 80 and the ratio of unexposed to exposed being 1. Using the open epi.com sample size calculation resource, a sample size of 90 participants was required. In consideration of potential loss to follow-up or incomplete data, 10% was added to this, giving an overall sample size of 99 participants. The structured interviewer-administered questionnaire was used to collect the data.

### 2.5 Data collection methods

Administration of questionnaires was done to collect quantitative data. The questionnaires had close-ended items. Six research assistants fluent in English, Rutooro and Runyankole, were selected and trained in data collection techniques and on how to administer the questionnaires.

Patients with abdominal trauma admitted in A&E of the two hospitals had history taking and physical examination, laboratory investigations and radiological investigation done by PI or study assistant. Patients, who met the clinical, laboratory and radiological criteria for laparotomy in accordance with the hospital care protocol in consultation with the attending surgeon, were consented. All patients were followed up post-operationally to assess for early outcomes.

At operation, the presence of bowel trauma was confirmed. After surgery, the participants were evaluated daily till the time of discharge and followed up weekly on phone calls for those staying far from the hospital and outpatient reviews for those staying nearer to the hospital.

### 2.6 Data analysis

The data obtained was summarized in excel sheets using Microsoft office excel. The summarized data was imported into STATA version 16. The percentage of patients with bowel trauma were computed as a fraction of all the patients operated for abdominal trauma and bivariate and multivariate analysis was done using binary logistic regression reporting both odds ratios and *P* values. Variables with *P* value ≤ 0.2 at bivariate were reanalyzed at multivariate using backward stepwise binary logistic regression. Variables with *P* ≤ 0.05 were considered significant factors associated with bowel trauma. Descriptive statistics was used to analyze complications and the number of complications plus percentages was reported. The percentage of patients with specific morbidities and mortality following was computed. A chi square test was used to compare the outcomes between those with bowel trauma and those without.

### 2.7 Ethical considerations

BSU REC (BSU REC-2023-243) provided ethical permission, and KIU-TH and FRRH provided administrative clearance. Standard forms in both local languages and English were used to acquire informed permission. There was no discrimination and participation was entirely voluntary. Data was anonymized and securely stored to guarantee confidentiality. There were no incentives offered, and no hazards beyond those of regular treatment were expected. The goal of the study is to enhance patient care in the future, even though participants did not directly benefit from it. At the research centers, all negative events were adequately managed.

## 3 Results

This study enrolled 99 study participants. According to the findings of this study, it was found that majority 72 (72.73%) of the respondents were aged between 20 and 49 years. Majority of the respondents 76 (76.77%) were males as detailed in Table 1 found below.

**Table 1:** Showing socio-demographic characteristics of the respondents at both sites.

Variable		Frequency (*N*)	Percentage (%)
Age group (Yrs)	<20	10	10.1
	20–49	72	72.7
	≥ 50	17	17.2
Sex	Male	76	76.8
	Female	23	23.2
Area of residence	Town	45	45.5
	Village	54	54.5
Distance from facility	<3 km	61.6	61.6
	>3 km	38.4	38.4

### 3.1 Causes of traumatic bowel injuries

The main cause of injury was stab wound 41(41.5%) followed by RTA with 31(31.3%) as shown in the Table 2 below.

**Table 2:** Causes of bowel injuries.

Cause of injury	Frequency (*N*)	Percentage (%)
Road Traffic/Accident	31	31.3
Gunshot	13	13.1
Stab wound	41	14.5
Falls	14	14.1

### 3.2 Anatomical patterns of traumatic bowel injury among patients with traumatic bowel injuries

This study revealed that small bowel was the most common site of traumatic bowel injury accounting for 76.5% (26/34) and especially the jejunum with 43% (Table 3).

**Table 3:** Anatomical patterns of traumatic bowel injury.

Variable		Frequency (*N*)	Percentage (%)
Injured Bowel *N* = 34	Small bowel	26	76.5
	Large	4	11.8
	Both	4	11.7
Part of injured small bowel *N* = 26	Duodenum	6	20.0
	Jejunum	13	43.3
	Ileum	9	30.0
	Jejunum & Ileum	2	6.7
Part of large bowel Injured *N* = 8	Sigmoid	5	62.5
	Transverse colon	3	37.5

### 3.3 Types of bowel surgery done among patients with traumatic bowel injuries

Majority of the patients had primary repair **(58.7%)** followed by **(20.6%)** resection and anastomosis and remaining had stomas as reported in the Table 4 below.

**Table 4:** Type of surgery done for the bowel injury.

Types of Surgery	SB	%	LB	%	TT	%
Primary Repair	15	44	5	14.7	20	58.7
Resection + Anastomosis	7	20.6	0	0	7	20.6
Loop ileostomy	4	11.76	0	0	4	11.76
Loop Colostomy	0	0	3	8.8	3	8.8
TOTAL	26	76.5	8	23.5	34	100

SB: Small bowel, LB: Large bowel.

### 3.4 Factors associated with traumatic bowel injury among patients undergoing surgery for abdominal trauma

In the bivariate analysis, the variables/factors who's any of its value that had a *p* value less than *P* ≤ 0.2 was considered for multivariate analysis these include residence, and history of alcohol. Detailed bivariate findings of socio-demographic factors are shown in Table 5 below.

**Table 5a:** Bivariate logistic regression of factors associated with traumatic bowel injury among patients undergoing surgery for abdominal trauma.

Variables	Bowel injury		cOR(95%CI)	*P*-Value
	Yes	No		
Age				
<20 years (1)	4	6	1.60 (0.31–8.25)	0.574
20–49 years	25	47	1.28 (0.40–4.03)	0.677
≥50 years	5	12	Ref.	
Sex				
Male	26	50	0.98 (0.37–2.59)	0.960
Female	8	15	Ref.	
Area of residence				
Town	19	26	1.90 (0.82–4.39)	0.134
Village	15	39	Ref.	
Distance from health facility (km)				
<3 km	24	37	Ref.	
≥3 km	10	28	0.55 (0.23–1.34)	0.187
History of smoking				
Yes	9	21	0.75 (0.30–1.89)	0.549
No	25	44	Ref.	
History of taking alcohol				
No	10	37	Ref.	
Yes	24	28	3.17 (1.31–7.69)	0.011

cOR, Crude Odds Ratio; CI, Confidence interval.

#### 3.4.1 Bivariate analysis of pre-operative and intra-operative characteristics associated with traumatic bowel injury among patients undergoing surgery for abdominal trauma

In the bivariate analysis, the variables/factors who's any of its value that had a *p* value less than *P* ≤ 0.2 was considered for multivariate with significance at with *p*-value of < 0.05 at 95% analysis these include mechanism of injury, any other abdominal organ, extra-abdominal organ injured, cause of injury, ASA score and HB. Detailed bivariate findings of socio-demographic factors are shown in Table 5b below.

**Table 5b:** Bivariate logistic regression of pre-operative and intra-operative characteristics associated with traumatic bowel injury study participants.

Variables	Bowel injury		cOR(95%CI)	*P*-Value
	Yes	No		
HIV				
Yes	2	6	0.62 (0.12–3.22)	0.565
No	32	59	Ref	
Hypertension				
Yes	2	4	0.95 (0.17–5.49)	0.957
No	32	61	Ref,	
Diabetes				
Yes	2	2	1.97 (0.27–14.63)	0.508
No	32	63	Ref.	
Mechanism of injury				
Blunt abdominal injury	12	37	Ref.	
Penetrative abdominal injury	22	28	2.42 (1.03–5.71)	0.013
Any other abdominal organ injury				
Yes	11	11	2.35 (0.89–6.18)	0.084
No	23	54	Ref	
Extra-abdominal organ injured				
Yes	1	9	0.19 (0.02–1.56)	0.121
No	33	56	Ref.	
Cause of injury				
Road Traffic/	10	21	0.86 (0.23–3.23)	0.825
Gun shots	1	10	0.15 (0.06–1.52)	0.108
Stab wounds	18	23	1.41 (0.40–4.94)	0.593
Falls	5	9	Ref.	
ASA score				
I	27	21	Ref.	
II	5	22	0.18 (0.06–0.55)	0.003
III	1	21	0.04 (0.01–0.29)	0.002
IV	1	1	0.78 (0.05–13.18)	0.862
Cadre of surgeon				
Intern	5	11	0.91 (0.16–5.19)	0.915
Medical officer	12	19	1.26 (0.27–6.03)	0.770
General surgeon	14	29	0.97 (0.21–4.44)	0.964
Consultant/specialist	3	6	Ref.	
Body Mass Index				
<18.5	11	14	1.53 (0.58–4.04)	0.393
18.5–24.9	18	35	Ref.	
25–29.9	4	11	0.71 (0.19–2.54)	0.595
≥30	1	5	0.39 (0.04–3.58)	0.405
Blood pressure				
Normal	28	58	Ref.	
Low BP	5	5	2.07 (0.55–7.75)	0.279
High BP	1	2	1.04 (0.09–11.91)	0.978
Temperature				
Normal	32	63	Ref.	
Low	1	2	0.98 (0.09–11.27)	0.990
High	1	0		
Pulse				
<60	29	62	Ref.	
60–100	3	2	3.21 (0.51–20.25)	0.215
>100	2	1	4.28 (0.37–49.09)	0.243
HB				
<10	4	1	8.53 (0.91–79.66)	0.260
10–18	30	64	Ref.	

cOR, Crude Odds Ratio; CI, Confidence interval.

Only participants with intestinal injuries were included; associated abdominal and extra-abdominal injuries are reported separately to clarify their relationship with bowel injury outcomes.

#### 3.4.2 Multivariate analysis to show factors independently associated with traumatic bowel injury among patients undergoing surgery for abdominal trauma

In the multivariate analysis, respondents that had penetrative injuries were 3.62 times most likely to sustain traumatic bowel injury (aOR: 3.62, 95%CI: 1.81–16.21, *P* = 0.048). On the other hand, Patients that had an ASA (American Society of Anesthesiology) score II (aOR: 0.12, 95%CI: 0.02–0.84, *P* = 0.033) and III (aOR: 0.01, 95%CI: 0.001–0.15, *P* = 0.002) were 0.12 times (88%) and 0.01 times (99%) less likely to have attained traumatic bowel injury compared to those that had ASA score I.F or details see the Table 6 below.

**Table 6:** Multivariate analysis to show factors independently associated with traumatic bowel injury among patients undergoing surgery for abdominal trauma.

Variables	Bowel injury		cOR(95%CI)	*P*-Value	aOR(95%CI)	*P*-Value
	Yes	No				
Area of residence						
Town	19	26	1.90 (0.82–4.39)	0.134	0.64 (0.11–3.68)	0.614
Village	15	39	Ref.		Ref.	
Distance from health facility (km)						
<3 km	24	37	Ref.		Ref.	
≥3 km	10	28	0.55 (0.23–1.34)	0.187	0.64 (0.09–4.43)	0.663
History of taking alcohol						
No	10	37	Ref.		Ref.	
Yes	24	28	3.17 (1.31–7.69)	0.011	2.09 (0.47–9.28)	0.329
Mechanism of injury						
Blunt abdominal injury	12	37	Ref.		Ref.	
Penetrative abdominal injury	22	28	2.42 (1.03–5.71)	0.013	3.62 (1.81–16.21)	0.048
Any other abdominal organ injury						
Yes	11	11	2.35 (0.89–6.18)	0.084	2.87 (0.49–16.53)	0.239
No	23	54	Ref		Ref.	
Extra-abdominal organ injured						
Yes	1	9	0.19 (0.02–1.56)	0.121	0.99 (0.04–23.81)	0.997
No	33	56	Ref.			
Cause of injury						
Road Traffic/	10	21	0.86 (0.23–3.23)	0.825	2.49 (0.29–20.78)	0.401
Gun shots	1	10	0.15 (0.06–1.52)	0.108	0.25 (0.01–9.03)	0.447
Stab wounds	18	23	1.41 (0.40–4.94)	0.593	6.99 (0.73–66.85)	0.091
Falls	5	9	Ref.		Ref.	
ASA score						
I	27	21	Ref.			
II	5	22	0.18 (0.06–0.55)	0.003	0.12 (0.02–0.77)	0.026
III	1	21	0.04 (0.01–0.29)	0.002	0.01 (0.001–0.14)	0.001
IV	1	1	0.78 (0.05–13.18)	0.862	3.49(0.01–86.03)	0.657

aOR, Crude Odds Ratio; CI, Confidence interval.

## 4 Discussion

In this study, the commonest injured bowel was the small bowel and the site most injured was the Jejunum. Most patients with bowel injuries reported to have sustained penetrative abdominal trauma. Similarly, Naqvi et al., (14) in India found traumatic perforation was most commonly seen in the small bowel and more specifically the jejunum accounting for 66% of the patients. On contrast, in our study, 72 patients presented with blunt trauma while 28 presented with penetrating injury. The fact that the small bowel was the most commonly injured in both studies could be because it covers a larger volume of the abdomen as opposed to the large bowel. Also, the anterior part of the abdomen that is least protected in the case of penetrating trauma, is in close proximity with the small bowel.

A study done by Julius O et al. in Nigeria revealed that the most frequently injured hollow viscous was the small bowel accounted for 11.8% (9). The small intestine was the site of injury most frequently (57.8%) encountered among all bowel injuries. Penetrating injuries occurred in 55.6% of those. Gunshot wounds (44.4%), auto accidents (37.8%), stab wounds (8.9%), falls (6.7%), and impalement (2.2%) were the leading causes of injury whereas in the current study, road traffic accidents (33.33%) and fights (25.25%) were the most common causes of abdominal trauma while falls accounted for 14.14% of the trauma as stab wounds led to 16.16% and gunshots to 11.11% of the trauma cases. Differences in study populations can explain the differences in the etiology.

More respondents that reported with penetrating abdominal trauma were found to be having traumatic bowel injury. Penetrating abdominal trauma has been reported to increase the risk of bowel injury as opposed to blunt abdominal trauma. This is in agreement with a systematic review by (15) who wrote that traumatic hollow viscous and mesenteric injury are not common in blunt abdominal trauma, but they exhibit a prevalence of approximately 1% and 17% in penetrating trauma. The fact that hollow organs can be compressed and are malleable makes them less likely to be injured in blunt trauma, however, with penetrating injuries, these hollow organs are at increased risk since they cover majority of the surface area of the abdominal cavity.

We also noted that victims who arrived and scored an ASA score of II were less likely to be having traumatic bowel injury than those with ASA score of IV. This is because, ASA IV patients often have severe cardiovascular. When the bowel is injured, the stool and other bowel content like bile are introduced into the peritoneal cavity. This introduction of bowel contents results in occurrence of peritonitis. The occurrence of peritonitis results in tachycardia and hypotension which may result in septic shock. Also, it is common to have the mesentery injured resulting in significant bleeding which may result in hypovolemia shock.

### 4.1 The anatomical patterns of traumatic bowel injuries among patients undergoing surgery for abdominal trauma at KIU-TH and FRRH

Bowel injuries following abdominal trauma are a significant concern in emergency surgery, with a wide variation in anatomical patterns. The findings from the provided table indicate that small bowel injuries are more common than large bowel injuries, with 76.5% of cases involving the small bowel. In comparison, large bowel injuries accounted for 11.8% and 11.7% involved both small and large bowel injuries. These findings are consistent with studies conducted in various trauma centers worldwide, which report that the small bowel is more susceptible to injury due to its anatomical position and mobility within the abdominal cavity (16).

Among small bowel injuries, the jejunum was the most frequently affected segment, accounting for 43.3% of cases, followed by the ileum (30.0%), duodenum (20.0%), and combined jejunum and ileum injuries (6.7%). This distribution aligns with previous research indicating that the jejunum and ileum are highly vulnerable to blunt and penetrating trauma due to their relative fixation points and exposure in the abdominal cavity (17). The duodenum, although less commonly injured, poses a significant surgical challenge due to its retroperitoneal location and proximity to vital structures (18).

Regarding large bowel injuries, the sigmoid colon was affected in 62.5% of cases, while the transverse colon accounted for 37.5%. Studies have shown that the sigmoid colon is more prone to injury, particularly from penetrating trauma, due to its relatively fixed position and proximity to the pelvis (19). The transverse colon, though less commonly injured, can be affected in high-impact blunt trauma and gunshot wounds (20).

These findings have important clinical implications for the management of traumatic bowel injuries. The predominance of small bowel injuries necessitates a high index of suspicion in cases of abdominal trauma, especially in patients with peritoneal signs or unexplained hemodynamic instability. Additionally, prompt surgical intervention is crucial for reducing morbidity and mortality associated with bowel perforation and peritonitis (21, 22).

In conclusion, the anatomical distribution of traumatic bowel injuries in the study is consistent with global patterns, highlighting the small bowel, particularly the jejunum and ileum, as the most commonly affected sites. The management approach should be tailored based on the site of injury, with an emphasis on early recognition, timely surgical intervention, and appropriate post-operative care.

### 4.2 Factors associated with bowel injury

More respondents that reported with penetrating abdominal trauma were found to be having traumatic bowel injury. Penetrating abdominal trauma has been reported to increase the risk of bowel injury as opposed to blunt abdominal trauma. This agrees with a systematic review by (8) who wrote that traumatic hollow viscous and mesenteric injury are not common inblunt abdominal trauma, but they exhibit a prevalence of approximately 1% and 17% in penetrating trauma. The fact that hollow organs can be compressed and are malleable makes them less likely to be injured in blunt trauma, however, with penetrating injuries, these hollow organs are at increased risk since they cover majority of the surface area of the abdominal cavity.

We also noted that victims who arrived and scored an ASA score of II were less likely to be having traumatic bowel injury than those with ASA score of IV. This is because, ASA IV patients often have severe cardiovascular. When the bowel is injured, the stool and other bowel content like bile are introduced into the peritoneal cavity. This introduction of bowel contents results in occurrence of peritonitis. The occurrence of peritonitis results in tachycardia and hypotension which may result in septic shock. Also, it is common to have the mesentery injured resulting in significant bleeding which may result in hypovolemic shock.

### 4.3 Strengths and limitations

This was the first study done in Uganda that focused on bowel injury reporting the factors associated with bowel injury among patients undergoing surgery for abdominal trauma. Our study was limited in that iatrogenic abdominal trauma was not evaluated in this study since majority of the iatrogenic injuries arise due to endoscopic procedures, which procedures are not done at the study centers from which patients were enrolled.

## 5 Conclusion

The commonest site injury was the small bowel with the jejunum being the most affected segment Female gender, mechanism of injury and the state of arrival of the patient at the health care facility were the significant factors associated with traumatic bowel injury. Furthermore, Primary repair and intestinal anastomosis were the most performed surgeries in Traumatic bowel injuries.

## 6 Recommendations

Strengthening paramedical services is required to enhance quick response to trauma victims in order to avoid complications and improve patient stability on arrival to the health facility.

There is a dire need in training all staff handling trauma patients about advanced life trauma support (ATLS) to prevent misdiagnosing bowel injuries which could to more serious complications.

## Data Availability

The original contributions presented in the study are included in the article/Supplementary Material, further inquiries can be directed to the corresponding author.

## References

[1] Baccoli A Manconi AR Caocci G Pisu S. Isolated jejunal perforation after blunt trauma. Report of three cases. G Chir. (2010) 31(4):167–70. 20444335

[2] Gad MA Saber A Farrag S Ellabban GM. Incidence, patterns, and factors predicting mortality of abdominal injuries in trauma patients. N Am J Med Sci. (2012) 4(3):129–34. 10.4103/1947-2714.93889 22454826 PMC3309620

[3] Dongo AE Kesieme EB Irabor DO Ladipo JK. A review of posttraumatic bowel injuries in ibadan. ISRN Surg. (2011) 2011:478042. 10.5402/2011/478042 22084759 PMC3200064

[4] Demetriades D Murray JA Chan L Ordoñez C Bowley D Nagy KK. Penetrating colon injuries requiring resection: diversion or primary anastomosis? An AAST prospective multicenter study. J Trauma. (2001) 50(5):765–75. 11371831 10.1097/00005373-200105000-00001

[5] McMahon KR Burns B Yarger JB Rashmi B. Intestinal trauma. In: StatPearls [Internet]. Treasure Island (FL): StatPearls Publishing (2026). Available online at: https://www.ncbi.nlm.nih.gov/books/NBK557624/?utm_source=chatgpt.com (Accessed 2023 May 22). 32491556

[6] Brownstein MR Bunting T Meyer AA Fakhry SM. Diagnosis and management of blunt small bowel injury: a survey of the membership of the American association for the surgery of trauma. J Trauma. (2000) 48(3):402–7. 10.1097/00005373-200003000-00006. 10744276

[7] Biffl WL Kaups KL Cothren CC Brasel KJ Dicker RA Bullard MK. Management of patients with anterior abdominal stab wounds: a western trauma association multicenter trial. J Trauma. (2009) 66(5):1294–301. 10.1097/TA.0b013e31819dc688 19430229

[8] Clemens MS Peace KM Yi F. Rectal trauma: evidence-based practices. Clin Colon Rectal Surg. (2018) 31(1):17–23. 10.1055/s-0037-1602182 29379403 PMC5787397

[9] Julius GO Olusoga OA Amarachuku CE Ige JT Ajibola DB Afolayan JM. Abdominal trauma in a semi-urban tertiary health institution. J Emerg Pract Trauma. (2018) 4(2):67–72. 10.15171/jept.2018.07

[10] Lüning TH Keemers-Gels ME Barendregt WB Rosman C. Colonoscopic perforations: a review of 30,366 patients. Surg Endosc. (2007) 21:994–7. 10.1007/s00464-007-9251-7 17453289

[11] Cobb WS Heniford BT Sigmon LB Hasan R Simms C Kercher KW. Colonoscopic perforations: incidence, management, and outcomes. Am Surg. (2004) 70(9):750–8. 10.1177/000313480407000902 15481289

[12] Faria GR Almeida AB Moreira H Barbosa E Correia-da-Silva P Costa-Maia J. Prognostic factors for traumatic bowel injuries: killing time. World J Surg. (2012) 36:807–12. 10.1007/s00268-012-1458-7 22350477

[13] Naqvi RH Singh G Saroch T Trakroo S. Traumatic gastrointestinal perforation following abdominal trauma: a study in tertiary care centre. Int Surg J. (2022) 9:1446–9. 10.18203/2349-2902.isj20221898

[14] Devi PS Manikantan GM Chisthi M. Gastrointestinal perforations: a tertiary care center experience. Int Surg J. (2017) 4(2):709–13. 10.18203/2349-2902.isj20170218

[15] Bonomi AM Granieri S Gupta S Altomare M Cioffi SP Sammartano F. Traumatic hollow viscus and mesenteric injury: role of CT and potential diagnostic–therapeutic algorithm. Updates Surg. (2021) 73(2):703–10. 10.1007/s13304-020-00929-w 33340338 PMC8005390

[16] Smyth L Bendinelli C Lee N Reeds MG Loh EJ Amico F. WSES Guidelines on blunt and penetrating bowel injury: diagnosis, investigations, and treatment. World J Emerg Surg. (2022) 17:13. 10.1186/s13017-022-00418-y 35246190 PMC8896237

[17] Goedecke M Kühn F Stratos I Vasan R Pertschy A Klar E. No need for surgery? Patterns and outcomes of blunt abdominal trauma. Innov Surg Sci. (2019) 4(3):100–7. 10.1515/iss-2018-0004 31709301 PMC6817729

[18] Diggs LP Gregory S Choron RL. Review of traumatic duodenal injuries: etiology, diagnosis, and management. Am Surg. (2023) 89(5):1989–96. 10.1177/00031348211065091 34974741

[19] De Robles MS Young CJ. Outcomes of primary repair and anastomosis for traumatic colonic injuries in a tertiary trauma center. Medicina (B Aires). (2020) 56(9):440. 10.3390/medicina56090440 J Emerg Med. (2018) 54(2):199–206. PMC7558995 32878038

[20] Cullinane DC Jawa RS Como JJ Moore AE Morris DS Cheriyan J. Management of penetrating intraperitoneal colon injuries: a meta-analysis and practice management guideline from the eastern association for the surgery of trauma. J Trauma Acute Care Surg. (2019) 86(3):505–15. 10.1097/TA.0000000000002146 30789470

[21] Hong SY Kim SH Kim KH. Blunt isolated small bowel perforation intervention: does a delay in management matter? Emerg Med Int. (2020) 2020:7478485. 10.1155/2020/7478485 32566306 PMC7292993

[22] Fakhry SM Brownstein M Watts DD Baker CC Oller D. Relatively short diagnostic delays (<8 hours) produce morbidity and mortality in blunt small bowel injury: an analysis of time to operative intervention in 198 patients from a multicenter experience. J Trauma. (2000) 48(3):408–15. 10.1097/00005373-200003000-00007 10744277

